# Optimum harvest maturity for *Leymus chinensis* seed

**DOI:** 10.1242/bio.017780

**Published:** 2016-05-11

**Authors:** Jixiang Lin, Yingnan Wang, Mingming Qi, Xiaoyu Li, Chunxue Yang, Yongcui Wang, Chunsheng Mu

**Affiliations:** 1 Alkali Soil Natural Environmental Science Center, Northeast Forestry University/Key Laboratory of Saline-Alkali Vegetation Ecology Restoration in Oil Field, Ministry of Education, Harbin 150040, China; 2 Key Laboratory of Vegetation Ecology of Ministry of Education, Institute of Grassland Science, Northeast Normal University, Changchun 130024, China; 3 Northeast Institute of Geography and Agroecology, Chinese Academy of Sciences, Changchun 130012, China; 4 Institute of Applied Ecology, Chinese Academy of Sciences, Shenyang 110016, China

**Keywords:** *Leymus chinensis*, Seed quality, Timely harvest, Seed colour, Weight

## Abstract

Timely harvest is critical to achieve maximum seed viability and vigour in agricultural production. However, little information exists concerning how to reap the best quality seeds of *Leymus chinensis*, which is the dominant and most promising grass species in the Songnen Grassland of Northern China. The objective of this study was to investigate and evaluate possible quality indices of the seeds at different days after peak anthesis. Seed quality at different development stages was assessed by the colours of the seed and lemmas, seed weight, moisture content, electrical conductivity of seed leachate and germination indices. Two consecutive years of experimental results showed that the maximum seed quality was recorded at 39 days after peak anthesis. At this date, the colours of the seed and lemmas reached heavy brown and yellow, respectively. The seed weight was highest and the moisture content and the electrical conductivity of seed leachate were lowest. In addition, the seed also reached its maximum germination percentage and energy at this stage, determined using a standard germination test (SGT) and accelerated ageing test (AAT). Thus, *Leymus chinensis* can be harvested at 39 days after peak anthesis based on the changes in parameters. Colour identification can be used as an additional indicator to provide a more rapid and reliable measure of optimum seed maturity; approximately 10 days after the colour of the lemmas reached yellow and the colour of the seed reached heavy brown, the seed of this species was suitable for harvest.

## Abstract (precis)

**Summary:** *Leymus chinensis* can be harvested at 39 DAPA based on changes in seed colour and a germination test. In actual agricultural production, harvesting is optimal 10 days after the colour of the lemmas reaches yellow and the seed reaches heavy brown.

## INTRODUCTION

*Leymus chinensis* is a perennial species of Poaceae, distributed in the Eastern region of the Eurasian steppe zone, the Northern and Eastern parts of the People's Republic of Mongolia, and the Northeast China Plain (Lin et al., 2011). It is an important fodder grass that can provide high protein and carbohydrate for grazing livestock. In addition, this plant is highly tolerant of the drought and saline-alkaline conditions in Northeast China (Huang et al., 2002).

A growing number of grasslands have been degraded due to overgrazing; however *Leymus chinensis* is considered as one of the most important plant species for grassland improvement in Northeast China (Liu and Qi, 2004; Yang et al., 1995). As a consequence of this, an increasing number of *Leymus chinensis* seeds are required by humans; however, low seed germination percentages induced by dormancy hinder to its use in the restoration of deteriorated grassland. Previous studies have proposed some methods to improve the percentage of seeds that germinate such as a low temperature treatment, different germinating beds, and an exogenous hormone treatment (Yi, 1994; Yi et al., 1997; Ma et al., 2008); however, most methods are difficult to apply in agricultural production. The optimum harvest time on seed germination is also very important. For the *Leymus chinensis* seed, the effect of harvest time has yet to be determined.

To reap good quality seed timely harvest is important to seed viability and vigour. Early harvesting may result in low yield and poor seed quality, whereas late harvesting may also result in poor seed quality due to changes in the weather (Bedane et al., 2006). Therefore, an increasing number of researchers have focused on determining the optimum harvest time of the seeds using factors such as seed colour, seed moisture content, and degree-days of growth (Elias and Copeland, 2001; Wang and Zhou, 2006).

*Leymus chinensis* has an indeterminate flowering habit and sets seed in different batches during the mature period. The lack of uniformity in flowering leads to non-uniform seed maturation, making it difficult to determine the optimum time to harvest good quality seeds and especially seeds with a higher germination percentage. Several studies have investigated the seed production of *Leymus chinensis* and the responses of seed germination to environmental changes and seed dormancy; however information on the optimum harvest maturity for this species is scarce, and the best way to harvest the highest quality seeds and enhance germination based on seed development is still not reported. Clear understanding of the most suitable harvest time for *Leymus chinensis* will enhance the seed quality and will assist in the harvesting of this species in agricultural production. The aims of this present work were to explore the indicators of optimum seed maturity and clarify the relationships between seed germination and development stage.

## RESULTS AND DISCUSSION

**Fig. 1.**
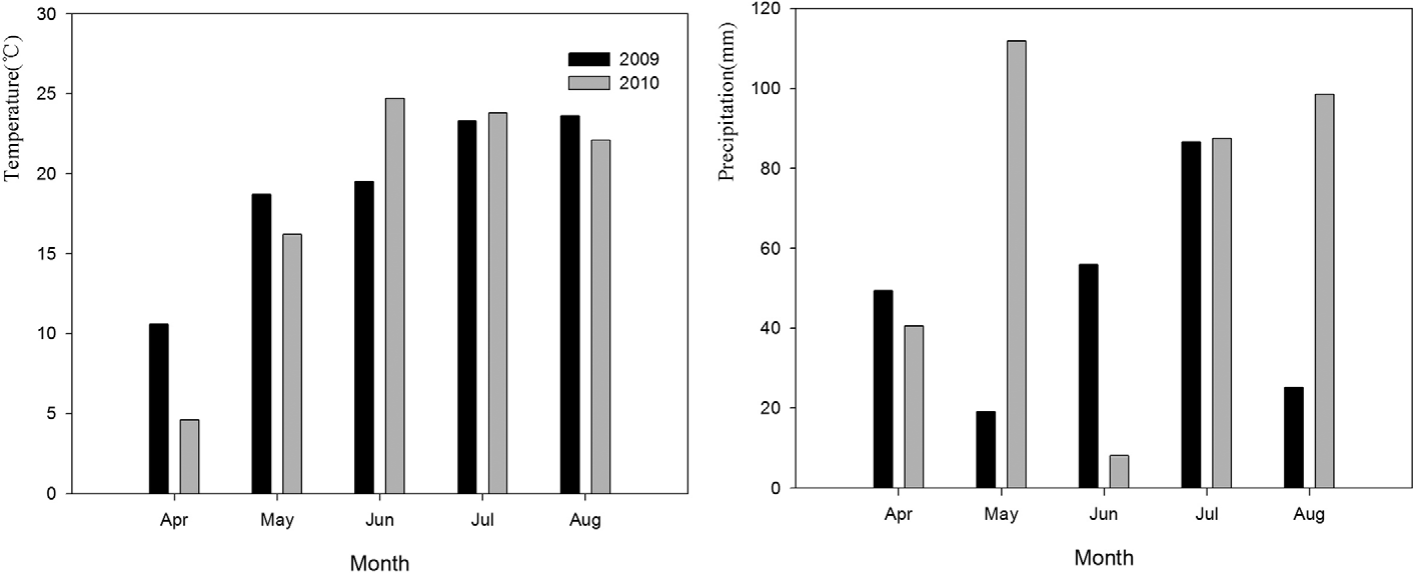
**Average daily temperature (°C) by month and precipitation (mm) by month for 2009 and 2010 in Songnen grassland of China.**

The average daily temperature from April to August (18.7°C and 18.2°C, respectively) in the two continuous sampling years was similar; however, the average daily temperatures for seed development stage in June and July 2009 (19.5°C and 23.3°C, respectively) were lower than those in the same period in 2010 (24.7°C and 23.8°C, respectively) (Fig. 1A). There was also a great difference in the average monthly precipitation between the two years, as it was higher in 2010 than in 2009 from April to August (Fig. 1B).

### Lemmas and seed colours

Gradual changes were found in lemmas and seed colours with seed development until maturation (Table 1). In the early stages of seed development, the lemmas was green from 6 to 18 days after peak anthesis (DAPA), then turned yellow green from 21 to 27 DAPA. In the final stage, the lemmas turned yellow from 33 to 48 DAPA. The seed colour was green from 6 to 12 DAPA, turned light green from 15 to 18 DAPA, and then heavy brown from 30 to 48 DAPA with the seed development.

**Table 1.**
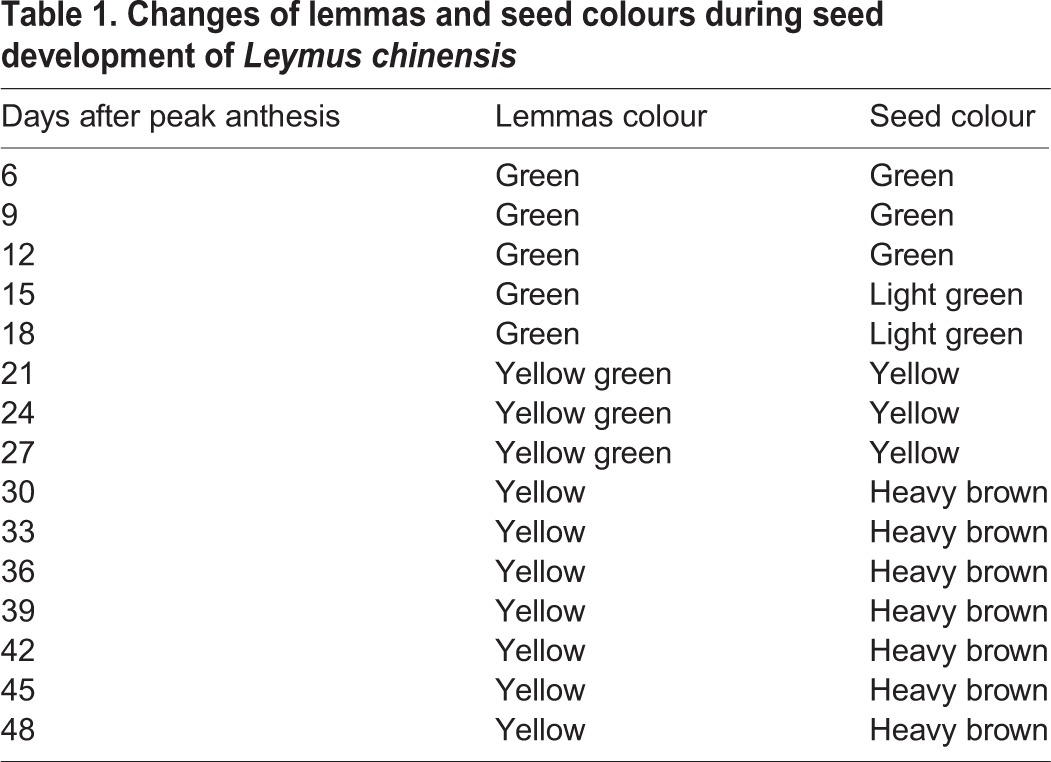
**Changes of lemmas and seed colours during seed development of *Leymus chinensis***

Visual indicators such as colour have been used as indicators of seed maturity and also to indicate the optimal harvest time (Miyajima, 1997; Wang et al., 2008). Physiological methods such as using the seed weight and seed quality to confirm the optimum harvest time are often time-consuming and difficult to perform in agricultural operations; however methods for determining the optimum harvest time for *L. chinensis* according to morphological indicators such as colour would minimize this constraint. In addition, using this method does not destroy the seeds and saves the germplasm resources. Previous studies have also demonstrated that seed colour offers an effective way of seed maturity in blume (*Trema micrantha* L.) (Castellani and Aguiar, 2001), buckwheat (*Fagopyrum esculentum* M.) (Funatsuki et al., 2000), and sesame seeds (*Sesamum indicum* L.) (Day, 2000). Changes in the colour of the lemmas and seed can provide a simple way for seed producers to judge the optimal harvest time.

### Seed weight

**Fig. 2.**
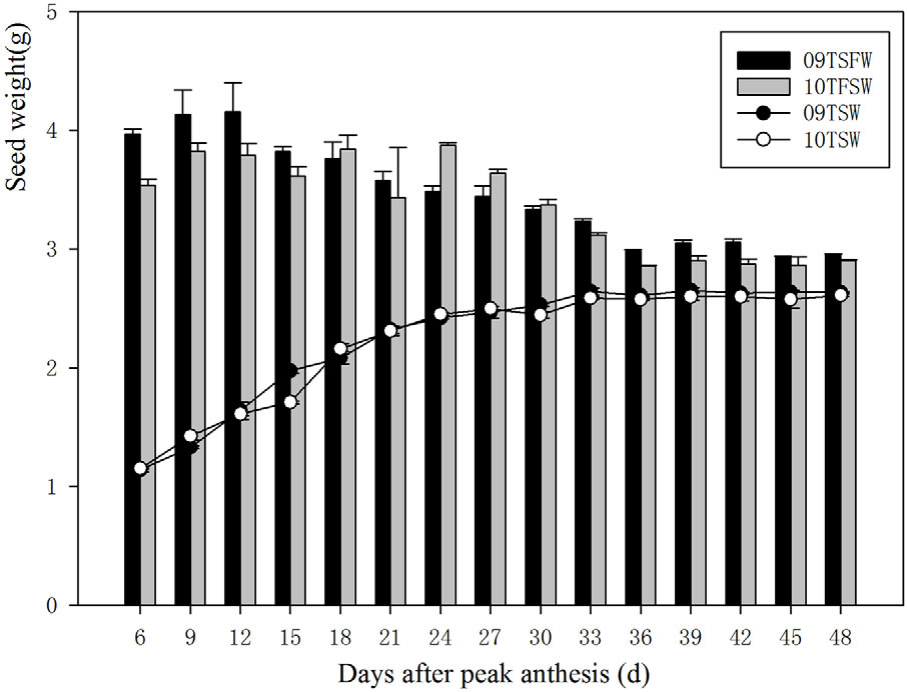
**Fresh weight (TSFW) and dry weight (TSW) of 1000 *Leymus chinensis* seeds during seed development in 2009 and 2010.** Seed weight (both TSFW and TSW) increased with seed development. The maximum 1000-seed weight over the two years was at 33 DAPA and did not change significantly with further seed development. Error bars indicate the standard error from means of TSFW and TSW at *P*=0.05 (*n*=8).

The changing rule of the 1000-seed weight of *L. chinensis* in 2009 was similar to 2010. The dry weight of *L. chinensis* seeds was significantly affected by the year, seed age and their interactions. Although the fresh weight was also significantly affected by the year and seed age, the influence of their interactions was not significant (Table 2). The seed weight increased with seed development, which was shown at 6-18 DAPA when the 1000-seed weight significantly increased due to the rapid accumulation of dry matter and the decreased moisture content (*P*<0.05). The maximum 1000-seed weight over the two years was at 33 DAPA and did not change significantly with further seed development (Fig. 2).

**Table 2.**
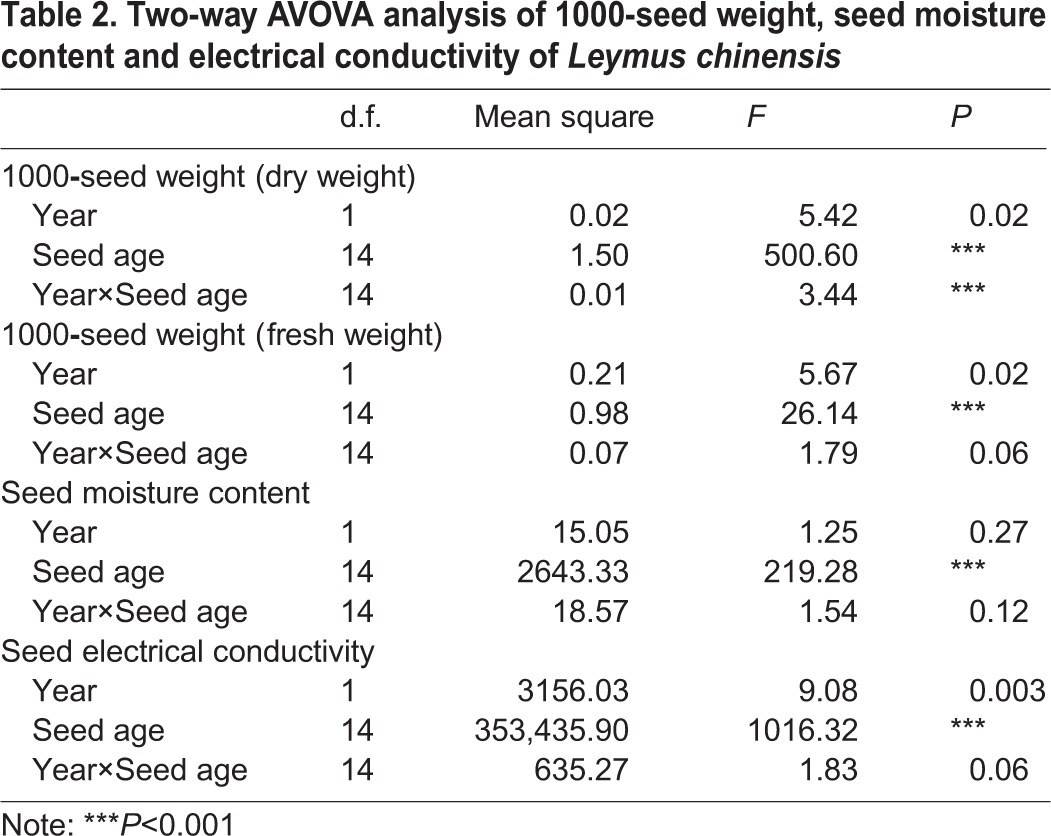
**Two-way AVOVA analysis of 1000-seed weight, seed moisture content and electrical conductivity of *Leymus chinensis***

Seed weight can directly reflect the extent of dry matter accumulation, and it is also used as an important indicator of seed quality and yield. In general, higher quality seeds always have higher seed weights. When the seed weight reaches a constant, the seed yield is always at the maximum. We found that the seed weight of the two experimental years both reached a maximum at 33 DAPA; however according to the germination test data, the germination quality at this stage was not maximum, so the seed weight can not be used as an indicator of the ideal seed harvest time of this species.

### Moisture content and electrical conductivity of seed leachate

**Fig. 3.**
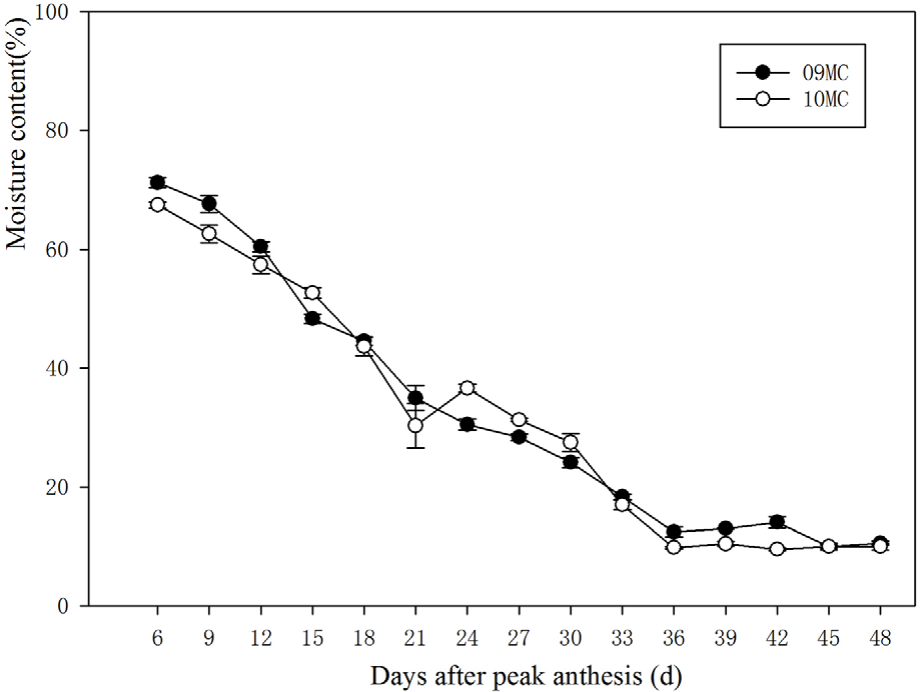
**Moisture content (%) of *Leymus chinensis* during seed development in 2009 (09MC) and 2010 (10MC).** The moisture content decreased with seed development, and the minimum appeared at 36 DAPA for both years. Error bars indicate the standard error from means of MC at *P*=0.05 (*n*=8).

**Fig. 4.**
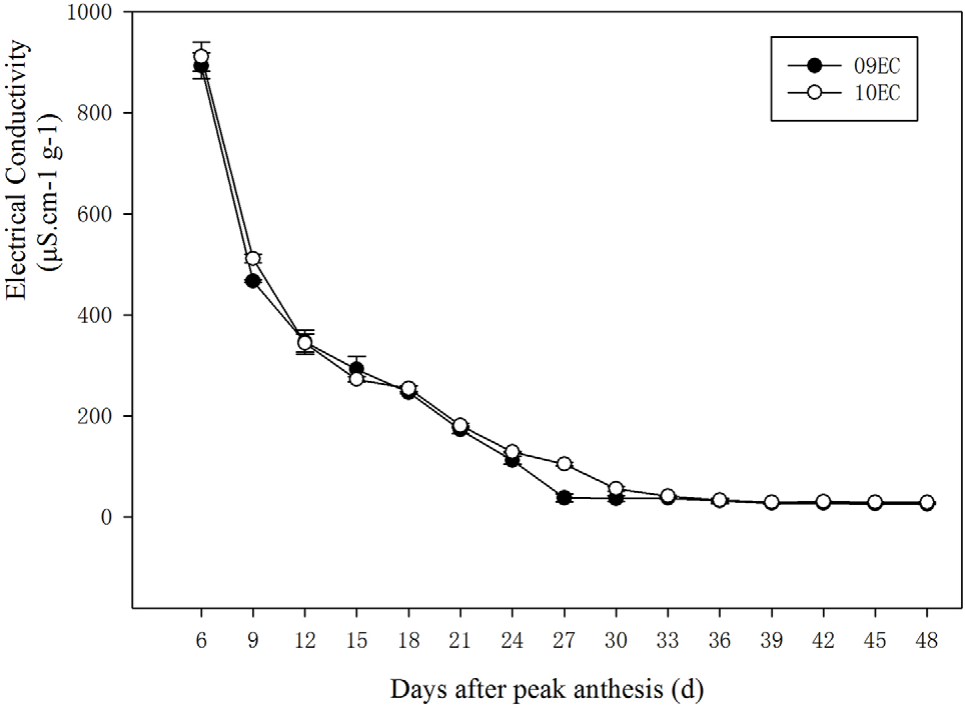
**Electrical conductivity of seed leachate of *Leymus chinensis* during seed development in 2009 (09EC) and 2010 (10EC).** Electrical conductivity (µS.cm^−1^ g^−1^) decreased with seed development, and reached a steady state at 27 and 30 DAPA over the two years. Error bars indicate the standard error from means of EC at *P*=0.05 (*n*=8).

The moisture content was also affected by seed age (Table 2). As the seed developed, the moisture content decreased and the minimum value appeared at 36 DAPA for both years (Fig. 3). In addition, the electrical conductivity of the seed leachate also decreased as the seed developed, especially at 6 and 9 DAPA. The value reached a steady state at 27 and 30 DAPA in the two experimental years. A two-way ANOVA showed that the electrical conductivity of the seed leachate was significantly affected by the year and the seed age (Table 2, Fig. 4).

With the increased seed weight, the seed moisture content decreased gradually. The seed moisture content has also been used as an index of the optimum harvest time (Abdelmotaleb, 1986; Andrade and Grabe, 2001; Castellani and Aguiar, 2001); however as opposed to other indices, the moisture content is always affected by changes in the environment such as long periods of sunny or rainy days. We found that the seed moisture content had the same problem as seed weight, as when the values of the two years reached a maximum the germination index was not at its highest point. Although seed moisture content is an important index, it also cannot be used as an indicator for the ideal seed harvest time of *Leymus chinensis*.

Seed vigour has a close relationship with the integrity of the cell membrane. The electrical conductivity of the seed leachate can describe the cell membrane well, and as a result it can also reflect the seed quality; however for some plants, the electrical conductivity of the seed leachate can not be used as an indicator for seed harvest, such as *Hordeum brevisubulatum* (Wang and Zhou, 2006). In our research, the electrical conductivity of the seed leachate reached minimum at 30 DAPA; however the seed weight was not very high and seed moisture content was still higher at this stage. In addition, germination percentages were only 68.9% and 63.3% in the two years. As a result, the electrical conductivity of the seed leachate can not be used as an indicator for the ideal seed harvest time of this species.

### Seed germination test

Great changes were found in seed germination across different sampling dates. There was no seed that could germinate at 6 to 12 DAPA as both the germination percentage and energy were 0. The germination percentage and energy (determined by SGT) enhanced markedly from 15 to 39 DAPA and then reached a steady state from 39 to 48 DAPA (77.8% and 75.6%) (Tables 3, 4). The germination percentage and energy determined by the AAT increased following peak anthesis, and the seed development time had no effect on the germination rate or energy from 39 to 42 DAPA. From germination test results, we can see that the seed germination reached a maximum at 39 DAPA (Tables 3, 4).

**Table 3.**
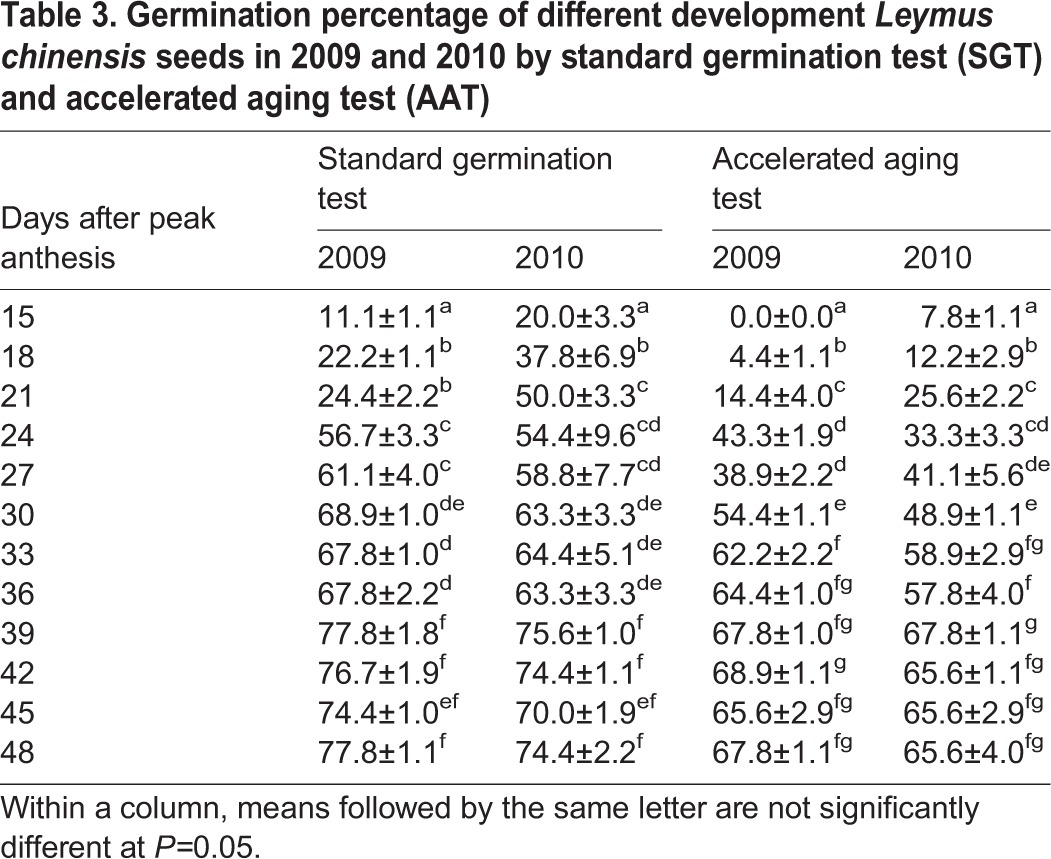
**Germination percentage of different development *Leymus chinensis* seeds in 2009 and 2010 by standard germination test (SGT) and accelerated aging test (AAT)**

**Table 4.**
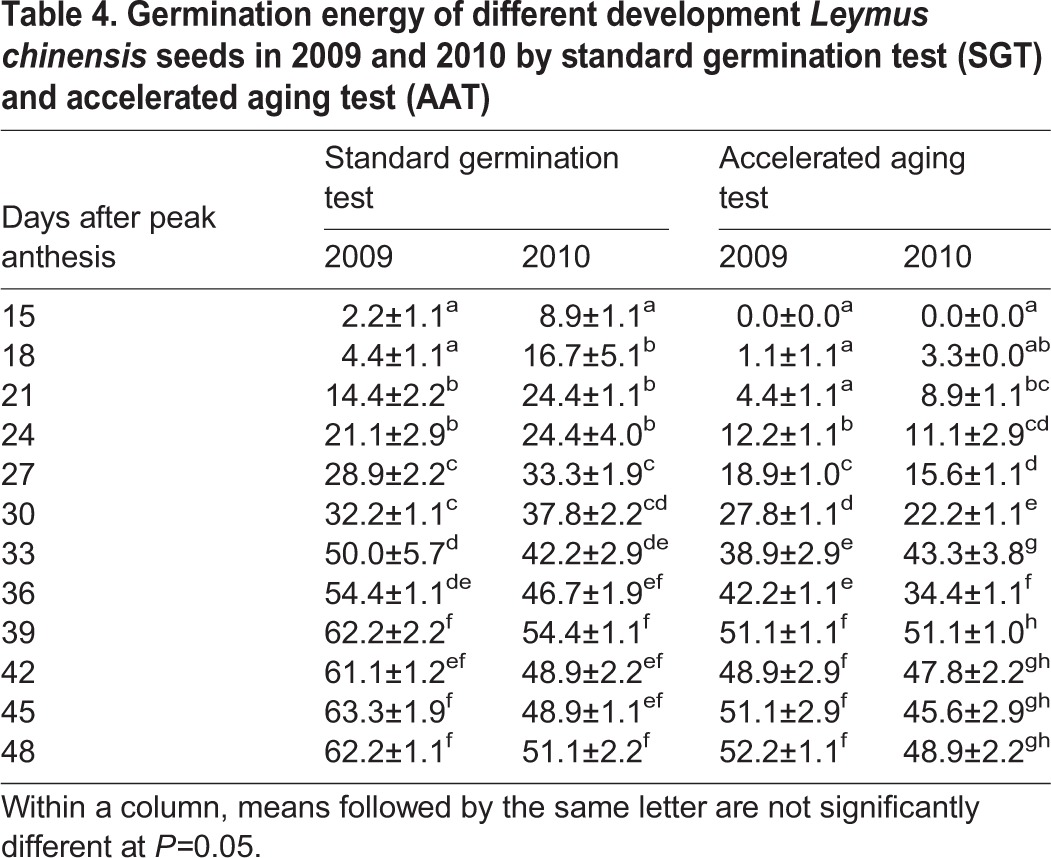
**Germination energy of different development *Leymus chinensis* seeds in 2009 and 2010 by standard germination test (SGT) and accelerated aging test (AAT)**

A two-way ANOVA showed that although the seed germination percentage and energy were not affected by the year, they were affected by seed age and the interactions between the year and seed age (Table 5).

**Table 5.**
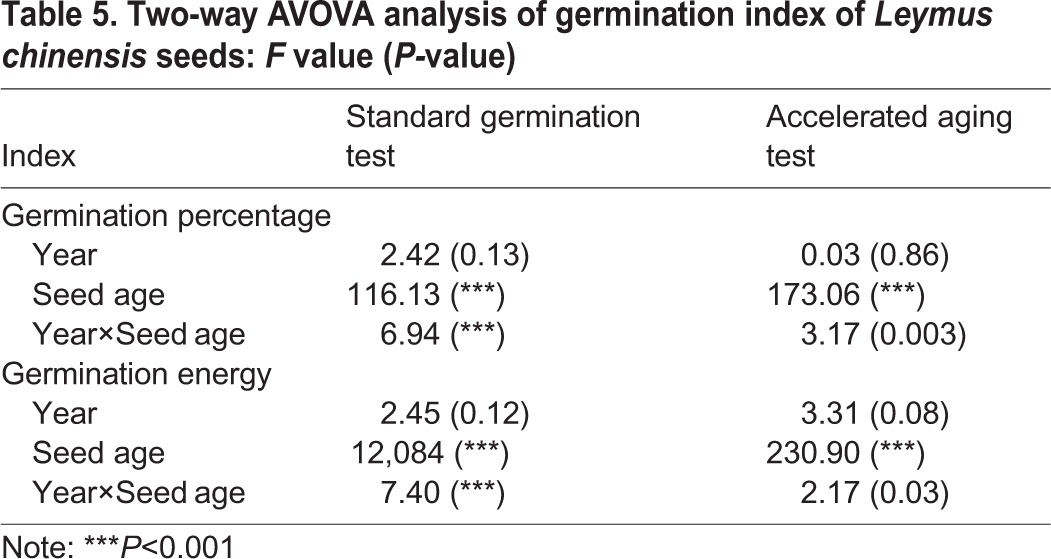
**Two-way AVOVA analysis of germination index of *Leymus chinensis* seeds: *F* value (*P-*value)**

The germination index is one of the most important indicators measuring seed quality, and it directly affects the emergence rate of seedlings in the field. In this study, the germination percentage and energy were both highest in SGT and AAT at 39 DAPA in the two years, indicating that seed vigour and quality were best at this stage (Tables 3, 4). Combined with the other indexes, such as the seed weight and seed moisture content, we found that 39 DAPA was ideal for the seed harvest of *Leymus chinensis*.

In general, grasses in the Poaceae have an indeterminate flowering habit and set seed in different batches during the mature period. In addition, once the seeds mature they fall off of the maternal plant. Although *Leymus chinensis* is not a threshing-type forage, adverse natural factors such as heavy wind or rain can greatly affect the seed yield and quality; therefore the determination of the optimum harvest time of *Leymus chinensis* in relation to seed quality during development is very important. According to our results in 2009 and 2010, *Leymus chinensis* can be harvested at 39 days after peak anthesis. In actual agricultural production, we can harvest the seeds based on the colours of the lemmas and seed. In summary, 10 days after the colour of the lemmas reaches yellow and the colour of the seed reaches heavy brown, the seeds are suitable for harvest.

## MATERIALS AND METHODS

### Experimental site and seed source

The experiment was conducted in a plot of 300 m^2^ located in the Ecosystem Field Station of the Institute of Grassland Science at the Songnen Grassland in the Jilin province of China (123°44′E, 44°44′N). This area is characterized by a semi-arid, continental monsoonal climate. The mean annual precipitation and temperature are 300-450 mm and 4.6°C to 6.4°C. The soil type is mixed salt-alkali meadow soil (Zhang et al*.*, 2009). The experiment was carried out on 10-year-old *Leymus chinensis* plants, from June to July in 2009 and 2010.

### Sampling procedures

Once flowering began daily field observations were conducted and all plants with similar flowering status were tagged when the majority of the plants in the plot flowered (dates of peak anthesis, DAPA). The sampling was conducted 6 days after peak anthesis and continued for 48 days in both of the experimental years, for a total of 15 sampling times in each year. The plants were clipped from the bottom of the stem by scissor every 3 days, and 80 plants were collected in every sample. Fifty plants were carefully put into separate nylon mesh bags and taken to the laboratory, while 30 plants were put into liquid nitrogen to determine the seed moisture content and fresh weight.

### Determination of 1000-seed dry weight and moisture content

The 1000-seed weight of all samples was determined with eight replicates and was calculated as the 100-seed weight×10 when the coefficient of variation was less than 6%. If the coefficient of variation was more than 6%, another eight randomly replicated samples were collected (Yan, 2000).

The moisture content was determined using eight replicates and was calculated as follows:
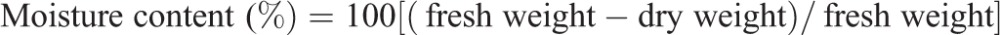


### Determination of the electrical conductivity (EC) of the seed leachate

The electrical conductivity (EC) of the seed leachate was tested according to the ISTA Handbook of Vigour Test Methods (Wellington, 1969). Four replicates of 100 weighted seeds were placed in four separate beakers with 100 ml deionized water at 20°C for 24 h, and the conductivity of the solution was measured.

### Germination test

A germination test was carried out in 11-cm petri dishes on two layers of filter paper moistened with 12 ml of distilled water. Four replicates of 50 seeds for each harvest were tested. The petri dishes were incubated at 20-30°C with a 16 h photoperiod (Sylvania cool white fluorescent lamps, 6400 lux) that coincided with the higher temperature in the growth chambers. Seeds were considered to be germinated with the emergence of the radicle. The germination percentage was recorded every 2 days for 20 days. The AAT was conducted by ageing seeds at 42°C for 72 h according to the ISTA Handbook (Wang et al*.*, 2008). The germination energy was calculated 7 days after seed germination.

### Statistical analysis

All data were subjected to analysis by the SPSS statistical software (version 13.0, SPSS Inc, Chicago, IL) and an analysis of variance (ANOVA). Two-way ANOVA were also used to analyse the seed weight, seed moisture content, and seed quality (electrical conductivity, germination test, and AAT, respectively) at the different sampling dates over the two sampling years. Differences in the different sampling dates were analysed using the least significant difference (LSD) test. The significance level was set at *P*≤0.05.
